# Tumor neoantigens and tumor immunotherapies

**DOI:** 10.1002/agm2.12295

**Published:** 2024-04-12

**Authors:** Wang Jiani, Tan Qin, Ma Jie

**Affiliations:** ^1^ Department of Biotherapy Center, Beijing Hospital, National Center of Gerontology Institute of Geriatric Medicine, Chinese Academy of Medical Sciences Beijing China

**Keywords:** cancer, neoantigen, tumor immunotherapy

## Abstract

As a high‐risk group of patients with cancer, the elderly exhibit limited efficacy with traditional treatments. Immunotherapy emerges as a promising adjunctive therapeutic approach that holds potential in addressing the needs of geriatric patients with cancer. Neoantigens, a unique class of tumor‐specific antigens generated by non‐synonymous mutations, are garnering increasing attention as targets for immunotherapy in clinical applications. Newly developed technologies, such as second‐generation gene sequencing and mass spectrometry, have provided powerful technical support for the identification and prediction of neoantigens. At present, neoantigen‐based immunotherapy has been extensively applied in clinical trials and has demonstrated both safety and efficacy, marking the beginning of a new era for cancer immunotherapy. This article reviews the conception, classification, inducers, and screening process of tumor neoantigens, as well as the application prospects and combination therapy strategies of neoantigen‐based cancer immunotherapy.

## Graphical abstract

Strategies for the identification, validation, and application of tumor neoantigens.

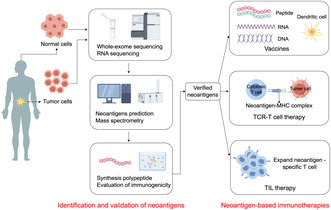

## 1 INTRODUCTION

There exists a significant correlation between senile diseases and cancer. The elderly are at high risk of cancer, and cancer demonstrates an escalating incidence and mortality rate with advancing age,^1^, ^2^ lies in several potential mechanisms. First, the DNA repair mechanism in older individuals may experience a decline, rendering them less capable of effectively repairing and countering gene mutations, thereby leading to an increased presence of abnormal cells and subsequent tumor formation.^3^ Second, the body's immune system gradually declines with age, even with immune surveillance disorders, which makes it difficult for the elderly to identify and eliminate abnormal cells and thus more likely to receive tumor invasion.^4^ Furthermore, the aggravation of chronic diseases in the elderly and the combined incidence of multiple diseases may also promote the occurrence of tumors. Undoubtedly, aging represents a pivotal risk factor for cancer development.

The treatment of elderly patients with tumors mainly includes surgery, radiation therapy , chemotherapy, and immunotherapy. However, many elderly patients with cancer have lost surgical indications at the time of diagnosis, and due to the degradation of physical function and the complexity of multiple chronic diseases, the effect and tolerance of radiation therapy and chemotherapy may be poor.^5^, ^6^, ^7^ Tumor immunotherapy is a promising complementary therapy for elderly patients with cancer by directly killing tumors and indirectly enhancing the killing of tumors by immune cells.^8^ Therefore, for the elderly patients with cancer, it is necessary to explore novel approaches for tumor treatments and develop individualized treatment strategies.

When tumors develop, the immune system identifies tumor cell surface antigens through precise regulation and monitoring functions, thereby initiating the body's specific humoral and cellular immune response, leading to the elimination of proliferating tumor cells. Tumor antigens can be divided into tumor‐associated antigens (TAAs) and tumor‐specific antigens (TSAs) according to the degree of their antigenic specificity.^9^ TAAs typically represent unmutated antigens that are overexpressed in malignant cells and are also expressed at low levels in normal cells, lacking strict tumor specificity.^10^ TSAs are neoantigens generated by non‐synonymous mutations and are exclusively expressed on the surface of certain tumor cells, with no expression in normal cells.^11^

## 2 CONCEPT AND CLASSIFICATION OF NEOANTIGENS

Neoantigens, arising from non‐synonymous mutations in the genome of tumor cells, encompass single nucleotide variants, insertion–deletion mutations, gene fusions, and structural variants.^12^, ^13^, ^14^ Compared to TAAs, neoantigens possess stronger immunogenicity and higher affinity toward major histocompatibility complex (MHC), remaining unaffected by central immunological tolerance. The immunogenicity enhances with the growing disparity between the mutant sequence and the normal sequence.^15^ Therefore, neoantigens emerge as ideal targets for therapeutic cancer vaccines and T cell‐based cancer immunotherapy.

Neoantigens can be categorized into two types: personalized neoantigens and shared neoantigens.^11^, ^14^ Personalized neoantigens pertain to the unique neoantigens of individual patients with cancer. Shared neoantigens are neoantigens that can be found in multiple patients with cancer but are not expressed in the normal genome. Personalized neoantigens can reduce the risk of immune escape of tumor cells due to their high immunogenicity. Personalized cancer vaccines made against them can be used independently or in combination with other therapies to increase the intensity and duration of antitumor effects. This approach aims to improve survival rates, quality of life, and ultimately improve the treatment outcomes for patients with cancer.^16^ Shared neoantigens play a crucial role in promoting tumor growth, most of which are oncogenic driver gene mutations. Examples include BRAF mutations in melanoma and KRAS mutations in pancreatic, colorectal, and endometrial cancers.^14^, ^17^, ^18^, ^19^ Due to the fundamental nature of driver mutations and their important role in tumor growth and metastasis, shared neoantigens are less likely to be lost as targets. Generic therapies based on shared neoantigens can save resources and time compared to personalized neoantigen therapies. Therefore, strategies targeting shared neoantigens have the potential and prospect of treating a broader patient population.^20^

## 3 FACTORS INDUCING NEOANTIGENS PRODUCTION

### 3.1 Chemotherapeutic drugs induce neoantigens

Chemotherapy constitutes a fundamental component of tumor treatment, which kills tumor cells by inhibiting their growth by way of suppressing actively dividing tumor cells. However, numerous preclinical studies have now demonstrated that, in addition to direct cytotoxic effects on cancer cells, a subset of DNA‐damaging agents may actually promote immunogenic alterations leading to increased neoantigenic loads, consequently activating antitumor immune responses.^21^ Alkylating chemotherapeutic agents, such as temozolomide and cyclophosphamide, which covalently modify DNA and induce cytotoxic damage to exposed cells, are respectively used to treat multiforme glioblastoma (GBM) and metastatic melanoma. Case reports have documented individual patients experiencing benefits from cancer vaccination following standard alkylating chemotherapy for GBM.^22^ These reports suggest that increased neoantigenic loads can enhance the effectiveness of chemotherapy combined with immunotherapy.

The form of neoantigen induced by chemotherapy drugs is named as drug‐induced xenogenization (DIX),^23^ such as the phenomenon in which the triazene compound temozolomide is used to promote point mutations of tumor cell generating neoantigens and cause tumor suppression. This xenogenization of tumor cells enhances their antigenicity and stimulates dendritic cells (DCs), prompting them to cross‐present tumor antigens to CD8^+^ T cells.^24^ Temozolomide demonstrates robust DIX properties compared to other chemotherapeutic agents. The study above suggests that the modulation of tumor mutational load by chemotherapeutic agents may open a new avenue for significantly enhancing the immunogenicity of malignant cells.^25^

### 3.2 Radiation therapy induces neoantigens

Radiation therapy works primarily through direct and indirect damage to cellular DNA through the action of radiation. Direct radiation‐induced damage destroys the structure of DNA molecules by causing single‐strand and double‐strand breaks, resulting in the death of mitotic cells within the radiation field. The mechanism of indirect damage involves the radiation‐induced radiolysis of water molecules, which generates unstable and unreactive hydroxyl radicals. These radicals then cause damage to DNA molecules and cell death.^26^ Radiation therapy cannot only mediate DNA damage‐induced cancer cell death, and trigger the release of pro‐inflammatory and anti‐inflammatory mediators to increase tumor‐infiltrating immune cells, but also regulate tumor immunogenicity and adjuvant properties by enhancing the expression of neoantigens.^27^

Research has shown that cancer neoantigens can be formed not only by genomic instability of the tumor itself but also by the effects of radiation on tumor cells. The immunogenic neoantigens produced during cancer radiation therapy create new immunotherapy opportunities for patients.^28^ Lhuillier et al^29^ reported that radiation therapy upregulated the expression of genes containing immunogenic mutations in a mouse model of triple‐negative breast cancer with low immunogenicity. Preparation of vaccines with new epitopes encoded by these genes induced CD8^+^ and CD4^+^ T cells and improved the therapeutic efficacy of radiation therapy.

### 3.3 Intratumoral microbes induces neoantigens

Based on examined whole‐genome and whole‐transcriptome sequencing studies in The Cancer Genome Atlas (TCGA) for microbial reads, unique microbial characteristics in tissues and blood have been observed in many types of cancer.^30^ A comprehensive analysis of the tumor microbiome found that each tumor type, including breast, lung, ovarian, pancreatic, melanoma, bone, and brain tumors, has a unique microbiome composition. Most of the bacteria are intracellular bacteria that exist in cancer and immune cells.^31^ With the discovery of tumor‐infiltrating microbiota, increasing evidence demonstrates the complex role of microbiota and host immunity in carcinogenesis.^32^, ^33^, ^34^, ^35^

Flora can directly or indirectly damage DNA by producing metabolites, such as genotoxic proteins and triggering specific immune responses, causing host gene mutations and increasing the risk of cancer.^36^ In 2020, Pleguezuelos‐Manzano et al^37^ published an article in the journal *Nature*, reporting that metabolites produced by intestinal microbes can directly cause gene mutations and lead to cancer. *Escherichia coli* (*E. coli*) carrying the pks virulence gene island can produce the gene toxin colibactin, resulting in DNA double‐strand breaks and host cell gene mutations. By long‐term co‐culture of human intestinal organoids with *E. coli*, researchers discovered that pks^+^ *E. coli* mainly caused two types of DNA mutations, single base substitutions and insertion–deletion mutations, compared with *E. coli* that did not produce colibactin. In this study, human intestinal organoids were used as a research model, which provided strong evidence for the association between *E. coli* and colorectal cancer from the perspective of gene mutation characteristics for the first time. Additionally, this study provided research support for the possibility of neoantigens induced by flora.

## 4 IDENTIFICATION AND VALIDATION OF NEOANTIGENS

The classical cDNA library screening method is a traditional approach for identifying tumor neoantigens.^38^ However, it has been gradually supplanted by next generation sequencing (NGS) due to time‐consuming and low throughput. Currently, the fundamental process of screening tumor neoantigens and identifying their immunogenicity includes the following steps: (1) obtaining a list of somatic mutations in the genome from whole‐exome sequencing data and combining it with the gene expression levels from transcriptome sequencing (RNA‐Seq) to initially screen for neoantigens. (2) Computer software was used to predict the affinity of tumor mutant peptides with MHC class I and II molecules, or mass spectrometry was used to identify the authentic epitope peptides bound to MHC molecules. (3) After identifying the candidate tumor neoantigen epitope, the corresponding polypeptide was synthesized, and the immunogenicity of the candidate tumor neoantigen was verified by immunological assays. Finally, it was applied to clinical trials such as therapeutic vaccines or T cell therapy based on tumor neoantigens.^39^, ^40^ However, due to the large number of screened candidate neoantigens, there are problems of fewer neoantigens capable of eliciting effective immune responses and higher rates of false‐positive predictions. As a result, the prediction results are often unsatisfactory. Therefore, by combining NGS with mass spectrometry and bioinformatics tools, the identification of neoantigens can be made more accurate.^14^ Mass spectrometry can help researchers directly analyze the MHC ligand group at the protein level using fewer tissue samples, and screen thousands of MHC‐presenting peptide sequences from cell lines and patient tissues to determine the real antigen epitopes.^41^ One of the important conditions for mass spectrometry to identify antigenic peptides is to obtain eluted MHC peptides. Immunoprecipitation is one of the most used methods to obtain eluted MHC peptides. The working process of mass spectrometry based on immunoprecipitation is as follows: immunoprecipitation of peptide–MHC complexes using magnetic beads embedded with MHC‐specific antibodies; then, gradually eluted to ensure that unbound and nonspecifically bound peptides are removed; subsequently, the eluent is subjected to mass spectrometry analysis, and finally, the actual protein library that can bind to MHC is obtained.^42^ Additionally, immunoproteomics based on mass spectrometry can better identify peptides that have undergone post‐translational modifications (such as phosphorylation,^43^ methylation, and glycosylation^44^) and spliced peptides produced by proteasomes. However, due to the large amount of tumor tissue involved in mass spectrometry, the pretreatment of samples is complicated, and the large number of identified antigen peptides, which ultimately affects its clinical application. At present, many research teams are working on the methodology of detecting neoantigens by mass spectrometry and have achieved significant results, which has greatly shortened the detection time.

## 5 NEOANTIGEN‐BASED IMMUNOTHERAPIES

Currently, immunotherapeutic approaches based on neoantigens generated by tumor gene mutations include therapeutic vaccines against tumor neoantigens, tumor neoantigen‐specific tumor‐infiltrating lymphocyte (TIL) therapies, and T cell receptor‐engineered T cell (TCR‐T) therapies, which are rapidly progressed from laboratory tests to clinical trials.

### 5.1 Neoantigen‐based vaccine

Neoantigen‐based vaccines represent an effective approach to activate and enhance antitumor T cell responses. The fundamental principle of this approach lies in promoting the proliferation and activation of neoantigen‐specific T cells in patients, to enhance the ability of the immune system to eliminate tumor cells while effectively preventing the autoimmune response induced by targeting TAA by traditional tumor vaccines. At present, the clinically applied neoantigen vaccines mainly include DC vaccines,^45^ peptide vaccines,^46^ nucleic acid vaccines,^47^, ^48^ which are administered subcutaneously or in lymph nodes. DC vaccines utilize DCs loaded with neoantigen peptides to present the neoantigens to T cells directly; peptide vaccines can be directly combined with the corresponding HLA on the DC cells in vivo after vaccination and is presented to the T cells; RNA vaccines are prepared by inoculating RNA fragments encoding new antigenic peptides, which are translated into new antigenic peptides in vivo and then presented by HLA.

Neoantigen‐based vaccines have achieved significant progress in various types of tumors, including malignant melanoma, glioma, lung cancer, gastric cancer, and pancreatic cancer, et al.^49^, ^50^, ^51^, ^52^, ^53^, ^54^, ^55^ The most representative study was reported in 2017 by the journal *Nature*, which described two successful cases of using neoantigen‐based personalized cancer vaccines to treat malignant melanoma. Ugur Sahin et al^49^ reported a study using an RNA vaccine against the corresponding neoantigens in 13 patients. All patients developed an immune response to the neoantigen following vaccination. Among them, the recurrence‐free survival time of eight patients with locally advanced tumors who underwent surgical treatment was significantly prolonged. Among the other five patients with advanced malignant melanoma, two patients experienced a significant reduction in tumor size and achieved objective remission after vaccination. Additionally, one patient achieved complete remission after receiving a vaccine combined with PD‐1 antibody treatment. Neoantigen vaccines have shown promising therapeutic effects against tumors in clinical trials and are expected to become an important tool for reducing tumor morbidity and mortality.

### 5.2 Neoantigen‐specific tumor‐infiltrating lymphocyte therapy

Tumor neoantigen‐specific TILs therapy is an anticancer treatment in which tumor neoantigen‐specific TILs are expanded in vitro and then infused back into the patient. Dudley et al^56^ were the first to use TILs as a method of adoptive immunotherapy, in which they isolated infiltrating T cells from surgically resected tumor tissues of the patient and infused them back into the patient after expansion in vitro by IL‐2 culture, in order to exert antitumor effects. In 2016, Tran et al^57^ discovered T cells targeting the KRAS^G12D^ mutation in a patient with metastatic colon cancer. After expanding and infusing these cells back into the patient, all seven lung metastases regressed, resulting in objective remission of metastatic colorectal cancer. Another study has shown that neoantigen‐specific response TILs can greatly enhance the sensitivity of tumor cells to immune checkpoint inhibitors, and the combination of the two can obtain a synergistic effect.^58^ The technology for culture and expansion of TILs is well‐developed, and when combined with neoantigen peptide libraries and other rapid neoantigen screening methods, neoantigen‐responsive TILs can be efficiently cultured, making them highly valuable for clinical applications.

### 5.3 Neoantigen‐specific TCR‐T cell therapy

TCR‐T is a therapeutic modality that uses genetic engineering to transfer TCRs that specifically recognize tumor antigens to autologous CD8^+^ T cells, giving them the ability to specifically kill tumor cells. Neoantigen‐specific TCR‐T cell therapy is a promising alternative treatment for individuals with weak antitumor immunity or a deficiency in tumor‐specific TILs.^59^ In 2022, Leidner et al^60^ reported a case of a patient with progressive metastatic pancreatic cancer who achieved objective remission of metastatic pancreatic cancer with 72% tumor shrinkage after TCR‐T therapy targeting KRAS^G12D^. Recently, a study on neoantigenic TCR‐T targeted therapy based on CRISPR technology was published in *Nature*, marking a significant advancement in the field of neoantigenic TCR‐T therapy. The researchers used CRISPR/Cas9 gene editing technology to genetically engineer T cells to identify mutant proteins that are unique to each patient's tumor. This targeted approach allows for the specific treatment of solid tumors, and this gene‐edited TCR‐T therapy is referred to as targeted neoantigen TCR‐engineered T cell product (neoTCR). In this study, 16 patients with different refractory solid tumors, including colon, breast, and lung cancers, were treated with up to three different individual‐specific neoTCR transgenic cell products. Out of these patients, five experienced tumor stabilization and their disease did not progress, whereas the remaining 11 patients had tumor progression. However, the study also confirmed that these modified cells do maintain higher concentrations near the patients' tumors, suggesting that the neoantigen TCR‐T is indeed better recognized and enriched around tumors.^61^ Neoantigen TCR‐T cell therapy has already shown promising results in treating solid tumors. To further enhance its therapeutic potential, efforts can be made to improve T‐cell homing, as well as increase the activity and persistence of infused T cells.

## 6 COMBINATION THERAPY STRATEGIES FOR NEOANTIGEN IMMUNOTHERAPIES

Immune checkpoint inhibitors have achieved remarkable success in cancer treatment by activating the host's immune system to eliminate tumors. However, the benefit of using immune checkpoint blockade as a single treatment option for most patients is limited.^62^ Comparative studies have shown that the combination of tumor vaccines and immunosuppressive therapies is more effective than monotherapy.^63^ The neoantigen vaccine stimulates the generation of T cells that are specific to the tumor and increases the expression of PD‐L1. This results in the production of more identifiable ligands for the immune checkpoint inhibitor PD‐L1 monoclonal antibody, leading to a synergistic antitumor response.^18^ According to the results of recent clinical trials, the combination of neoantigens and checkpoint inhibitors has been shown to enhance the overall survival rate of patients.^64^, ^65^

Traditional therapies, such as radiation therapy and chemotherapy, can also enhance the effects of neoantigen vaccines. When the number of neoantigen is too little to activate T cell response, the problem can be addressed by combining neoantigen vaccines with chemotherapy and radiation therapy. Studies have shown that chemotherapy or radiation therapy can induce tumor cells to release more antigens, and radiation therapy promotes T cell aggregation toward tumor tissue.^66^ Neoantigen‐specific CD8^+^ T cells preferentially kill irradiated tumor cells, providing evidence that the combination of radiotherapy and neoantigen immunization can be effective in controlling tumors.^29^ Chemotherapy enhances the effectiveness of neoantigen vaccines through mechanisms such as inducing immunogenic cell death, triggering cascade reactions, generating neoantigens, and facilitating phagocytosis and antigen presentation by dendritic cells.^67^, ^68^, ^69^ Temozolomide, for example, is primarily used to treat malignant brain tumors and is commonly prescribed as the initial treatment for glioblastoma. In the clinical trial NCT00905060, the combination of a neoantigen vaccine and temozolomide were tested for treating patients with brain and central nervous system tumors. The study found that the median overall survival was 23.8 months, and the median progression‐free survival was 18 months.^70^ These results suggest that combining the neoantigen vaccine with standard therapy, such as temozolomide, is crucial. This combination has the potential to improve the survival rate in patients with glioblastoma.

Furthermore, another study has demonstrated the synergistic effect of combining radio‐frequency ablation (RFA) in interventional therapy with vaccination using neoantigenic peptide vaccines for tumor treatment. Patients treated with RFA prior to personalized cancer vaccination showed a better clinical response, with a longer median progression‐free survival and median overall survival after neoantigen vaccination (2.82 months vs 4.42 months; and 10.94 months vs 20.18 months). The mouse model further validated the synergistic mechanism of the combination therapy of RFA and neoantigen vaccine.^71^

## 7 PROSPECTS AND CHALLENGES

With the global population aging, the incidence and mortality of tumors are increasing. Elderly patients with cancer face elevated mortality risks due to their intricate physiological conditions and unique disease progression patterns. It is urgent to explore tumor treatment strategies suitable for the elderly. At present, immunotherapy based on tumor neoantigens has played a huge role in clinical trials and is expected to become an important means to reduce cancer morbidity and mortality.

Tumor neoantigens have emerged as a prominent area of research in the field of immunotherapy and are expected to be a key focus of clinical immunotherapy in the future. However, the development of neoantigens faces many constraints, and the following problems must be solved to promote neoantigens better: (1) the prediction accuracy of neoantigens remains suboptimal. The quality of neoantigens is fundamental to determining whether cancer vaccines can effectively stimulate anti‐tumor immune responses. Due to variations in technology platforms, there are disparities in calculation methods, which result in inconsistent predictions. (2) The absence of cost‐effective methods for screening neoantigens is a barrier to the advancement of neoantigen therapies. Owing to the heterogeneity of tumors, tumor neoantigen vaccines are typically personalized for each individual recipient. It is due to the high degree of personalization that the preparation cycle for tumor neoantigen vaccines is very long and expensive to manufacture. (3) Although the review mentions several ways in which neoantigen production can be induced, the available research is still not sufficiently advanced. If patients treated with the same chemotherapeutic agents can generate shared neoantigens, the expense of neoantigen therapy can be decreased, and a comprehensive treatment strategy can be formulated for patients.

We maintain that the aforementioned problems can be well solved in the development of emerging detection technologies and the intersection and integration of bioinformatics, artificial intelligence, machine learning, and other related disciplines. The comprehensive research and resolution of the aforementioned issues will establish a solid groundwork for the clinical implementation of neoantigen therapy. We anticipate that neoantigens‐based therapies will assume an essential and significant role in the treatment of tumors in the future and create a more appropriate therapeutic effect for elderly patients with cancer.

## AUTHOR CONTRIBUTIONS

Formulated the research question and critically reviewed the article for intellectual content: Ma. Guided the thinking and review of the manuscript: Tan. Conducted the study, collected the information, drafted the manuscript, and drew the image by Figdraw: Wang.

## FUNDING STATEMENT

National High Level Hospital Clinical Research Funding (BJ‐2022‐118); National Natural Science Foundation of China (Grant No. 82003264).

## CONFLICT OF INTEREST STATEMENT

The author declares no relevant financial or non‐financial interests to disclose.
